# Supplementary material for: Spike-Dependent Opsonization Indicates Both Dose-Dependent Inhibition of Phagocytosis and That Non-Neutralizing Antibodies Can Confer Protection to SARS-CoV-2
Source: Front Immunol. 2022 Jan 14;12:808932. doi: 10.3389/fimmu.2021.808932 (PMC8796240; doi:10.3389/fimmu.2021.808932)
Supplement: Supplementary file 1 [file DataSheet_1.pdf]

Supplementary Figure 1

a

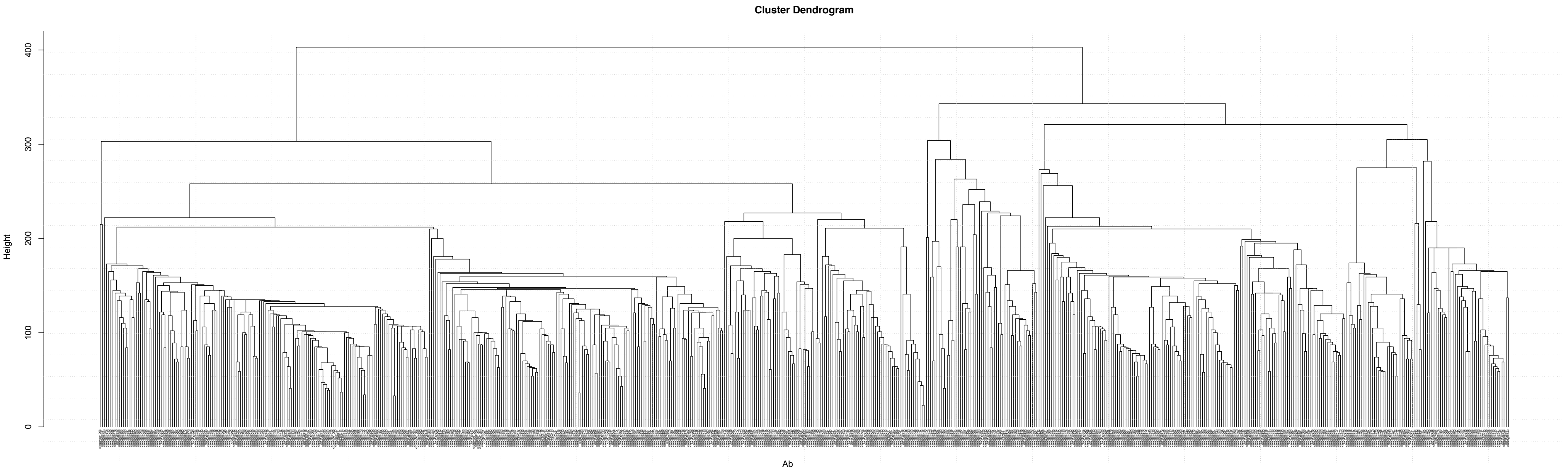

b

```
H7         EVQLVQSGAEVKKPGESLKISCKGSGYS-FS-TYWIGWVRQMPGKGLEWMGIIYPRDSET   58
H11        QVTLKESGPVLVKPTETLTLTCTVSGFSLSNAKMGVSWIRQPPGKALEWLAHI-FSNDEK   59
H36        QVQLVQSGAEVKKPGASVKVSCKASGYT-FT-SHAVHWVRQAPGQRLEWMGWINAGNGNT   58
H57        QVTLRESGPALVRPTQTLTLTCTFSGFSLSTSGMCVSWIRQPPGKALEWLARI-DWDDDK   59
H59        QVQLVQSGAEVKKPGASVKVSCKASGYT-FT-SYDINWVRQATGQGLEWMGWMNPNSGNT   58
H66        EVQLVESGGGLVKPGGSLRLSCAASGFT-FS-SYSMNWVRQAPGKGLEWVSSISSSSSYI   58
H77        QVQLVESGGGVVQPGRSLRLSCAASGFT-FS-SYDMHWVRQAPGKGLEWVALISYDGSNT   58
H81        QVQLVQSGAEVKKPGASVKVSCKASGYT-FT-SYGISWVRQAPGQGLEWMGWISAYNGNT   58
H94        QVQLQESGPRLVKPSGTLSLTCAVSGGSISS-SNWWTWVRQPPGKGLEWIGEI-YHSGST   58
H95        EVQLVESGGGLVQPGGSLRLSCVASGFI-VS-GNYLSWVRQAPGKGLEWVSVI-YSGGGT   57
           :* * :**  : :*  :: ::*  **     .      *:**  *: ***:. :   ..

H7         RYSPSFQGQITMSADRSISTAYLQWNSLKASDTAMYYCA-----------RWRSGWYLDL   107
H11        SYSTSLKSSLTISKDTSKSQVVLTMTNMDPVDTATYYCARLLW---------FGGNYFDY   110
H36        KYSQKFQGRVTITRDTSASTAYMELSSLRSEDTAVYYCARDPV--LRYF-DWTTPYYFDY   115
H57        YYSTSLRTRLTISKDTSKNQVVLTMTNMDPVDTATYYCARMTV-----------TTAFDI   108
H59        AYAQKFQGRVTMTRNTSISTAYMELSSLRSEDTAVYYCARGGR--YCSSTSCYSGVPNDY   116
H66        YYADSVKGRFTISRDNAKNSLYLQMNSLRAEDTAVYYCARRSFVSSST-DDYYYYYDMDV   117
H77        YYADSVKGRFTISRDNSKNTLYLQMNSLRAEDTAVFYCAKTIYSYALK-PNYF-----DY   112
H81        NYAQKVQGRVTMTRDTSTSTAYMELRSLRSDDTAVYYCARDGI--AVAE-DW-----FDP   110
H94        NYTPSLKSRVTISVDKSKNQFSLRLNSVTAADTAVYYCARGWS---------SSWYGLDY   109
H95        YYADSVKGRFTISRDNSKNTLYLQMNSLRAEDTAVYYCARDLLGGYCSGGSCKEGFYFAY   117
            *:  ..:  .*:: : : .   :   .:   *** :***

H7         WGRGSLVTVSS    118
H11        WGQGTLVTVSS    121
H36        WGQGTLVTVSS    126
H57        WGQGTMVTVSS    119
H59        WGQGTLVTVSS    127
H66        WGQGTTVTVSS    128
H77        WGQGTLVTVSS    123
H81        WGQGTLVTVSS    121
H94        WGQGTLVTVSS    120
H95        WGQGTLVTVSS    128
           **:*: *****
```

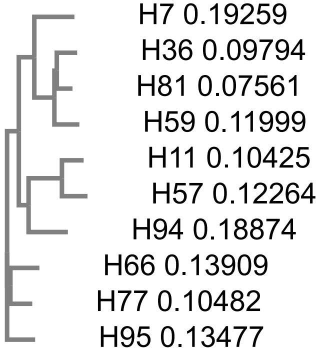

c

```
L7         QSALT-QPASVSGSPGQSITISCTGTSSDVGGYTHVSWYQQHPGKALKLMISEVSDRPSG   59
L11        SYELT-QPLSVSVALGQTARITCGGNNI---GSK-MHWYQQKPGQAPVLVIYRDSNRPSG   55
L36        EIVLTQSPGTLSLSPGERATLSCRASQS--FSSSYLAWYQQKPGQAPRLLIYGASSRATG   58
L57        DIQMTQSPSSLSASVGDRVTITCRASQS--I-SSYLNWYQQKPGKAPKLLIYAASSLQSG   57
L59        DIQLTQSPSFLSASVGDRVTITCRASQG--V-SSYLAWYQQKPGKAPKLLIYPASTLQSG   57
L66        SYELT-QPPSVSVSPGQTARITCSGDAL---PKQYAYWYQQKPGQAPVLVIYKDSERPSG   56
L77        DIQMTQSPSSLSASVGDRVTITCQASQD--I-SNYLNWYQQKPGKAPKLLIYDASNLETG   57
L81        SYELT-QPPSVSVAPGKTARITCGENNI---GSKSVHWYQQKPGQAPVLVIYYDSDRPSG   56
L94        QSALT-QPASVSGSPGQSITISCTGTSSDVGSYNLVSWYQQHPGKAPKLVIYEATKRPSG   59
L95        SYVLT-QPPSVSVAPGKTARITCGGNNI---GSKSVRWYQQKPGQAPVLVIYYDSDRPSG   56
           .  :* .*  :* : *.   ::*                ****:**:*  *:*   :   :*

L7         VSNRFSGSKSGSTASLTISGLQAEDEAHYYCSSYTSG--NTLVFGGGTKLTVL  110
L11        IPERFSGSNSGNTATLTISRAQAGDEADYYCQVWDSST---VVFGGGTKLTVL  105
L36        IPDRFSGSGSGTDFTLTISRLEPEDFAVYYCQHYGSS---PLTFGGGTKVEIK  108
L57        VPSRFSGSGSGTDFTLTISSLQPEDFATYYCQQSYST---PRTFGQGTKVEIK  107
L59        VPSRFSGSGSGTEFTLTISSLQPEDFATYYCQQLNSY---PLTFGGGTKVEIK  107
L66        IPERFSGSSSGTTVTLTISGVQAEDEADYYCQSADSSG-TYVVFAGGTKLTVL  108
L77        VPSRFSGSGSGTDFTFTISSLQPEDIATYYCQQYDNL---PPTFGGGTKVGIK  107
L81        IPERFSGSNSGNTATLTISRVEAGDEADYYCQVWDGSSDH-VVFGGGTKLTVL  108
L94        VSNRFSGSKSGNTASLTISGLQAEDEADYYCYSYAGS--STWVFGGGTKLTVL  110
L95        IPERFSGSNSGNTATLTISRVEAGDEADYYCQVWDSSSDHPWVFGGGTKLTVL  109
           : .***** **.  ::***  :  * * ***    .     .*. ***: :
```

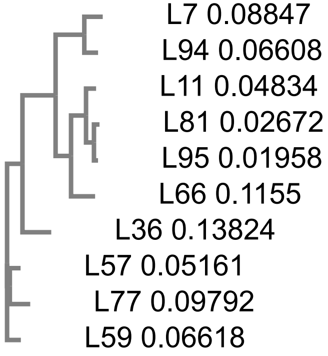

d

antibody	germline	CDR3 loop
Ab7	IGHV5-51*01	ARWRSGWYLDL
Ab11	IGHV2-26*01	ARLLWFGGNYFDY
Ab36	IGHV1-3*01	ARDPVLRYFDWTTPYYFDY
Ab57	IGHV2-70*15	ARMTVTTAFDI
Ab59	IGHV1-8*02	ARGGRYCSSTSCYSGVPNDY
Ab66	IGHV3-21*04	ARRSFVSSSTDDYYYYYDMDV
Ab77	IGHV3-33*03	AKTIYSYALKPNYFDY
Ab81	IGHV1-18*04	ARDGIAVAEDWFDP
Ab94	IGHV4-4*02	ARGWSSSWYGLDY
Ab95	IGHV3-53*02	ARDLLGGYCSGGSCKEGFYFAY

Supplementary Figure 1. Sequence analysis of Spike-reactive antibodies

a The plot shows the result of hierarchical clustering of the light-chain and heavy-chain sequences of the clonotypes visualized as an unrooted dendrogram. **b** and **c** Heavy chain (b) and Light chain (c) sequence alignment (Clustal Omega) for the 10 Spike-reactive antibodies seen with ELISA. Phylogenetic analysis of the variable regions is also shown comparing the heavy and light chains. **d** Germline family distribution and CDR3 sequences for all 10 Spike-reactive antibodies.
