# Supplementary material for: Spike-Dependent Opsonization Indicates Both Dose-Dependent Inhibition of Phagocytosis and That Non-Neutralizing Antibodies Can Confer Protection to SARS-CoV-2
Source: Front Immunol. 2022 Jan 14;12:808932. doi: 10.3389/fimmu.2021.808932 (PMC8796240; doi:10.3389/fimmu.2021.808932)
Supplement: Supplementary file 2 [file DataSheet_2.pdf]

Supplementary Figure 2

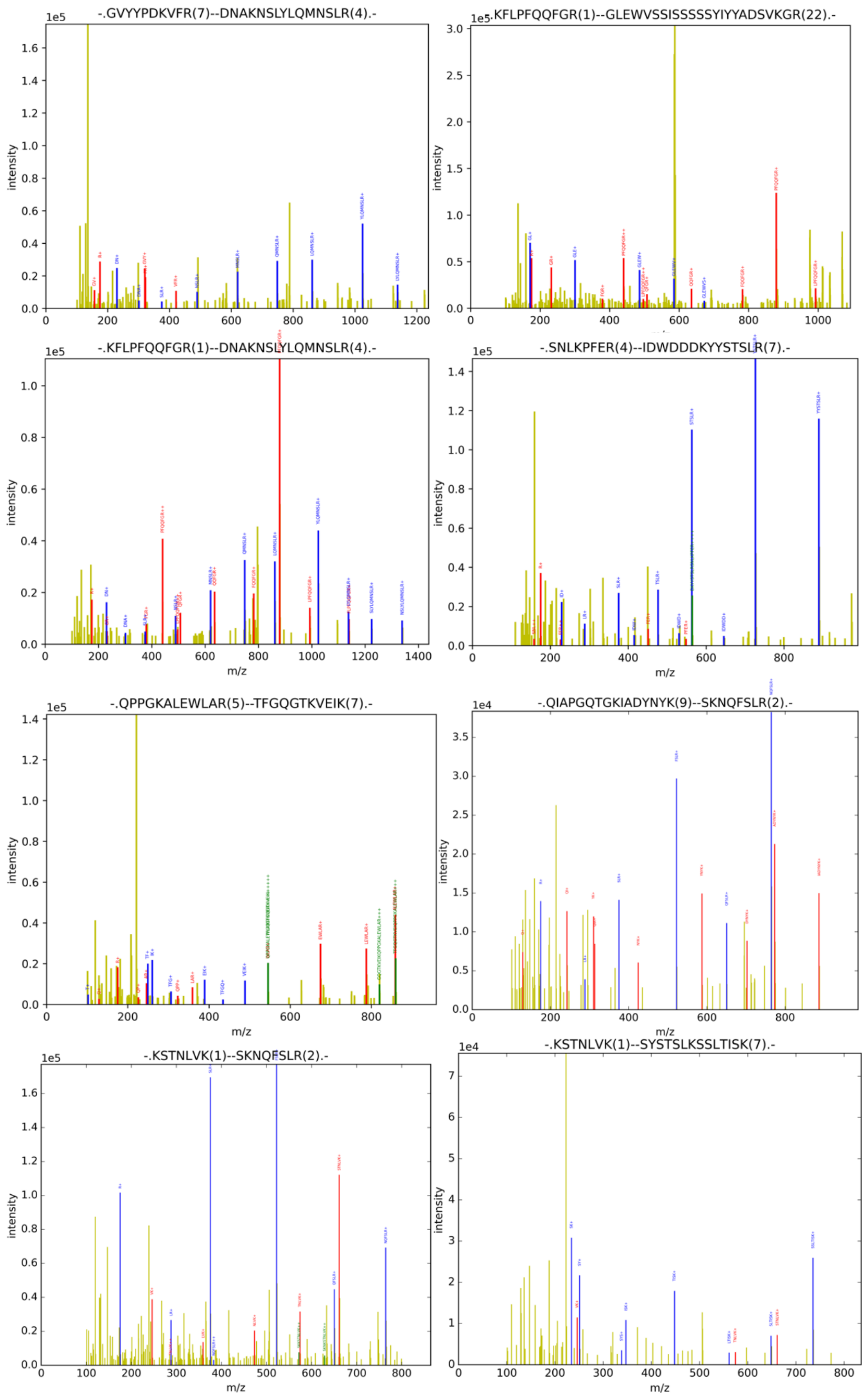

Supplementary Figure 2. MS spectra from Spike-antibody cross-linking experiments.
