# Supplementary material for: Spike-Dependent Opsonization Indicates Both Dose-Dependent Inhibition of Phagocytosis and That Non-Neutralizing Antibodies Can Confer Protection to SARS-CoV-2
Source: Front Immunol. 2022 Jan 14;12:808932. doi: 10.3389/fimmu.2021.808932 (PMC8796240; doi:10.3389/fimmu.2021.808932)
Supplement: Supplementary file 3 [file DataSheet_3.pdf]

Supplementary Figure 3

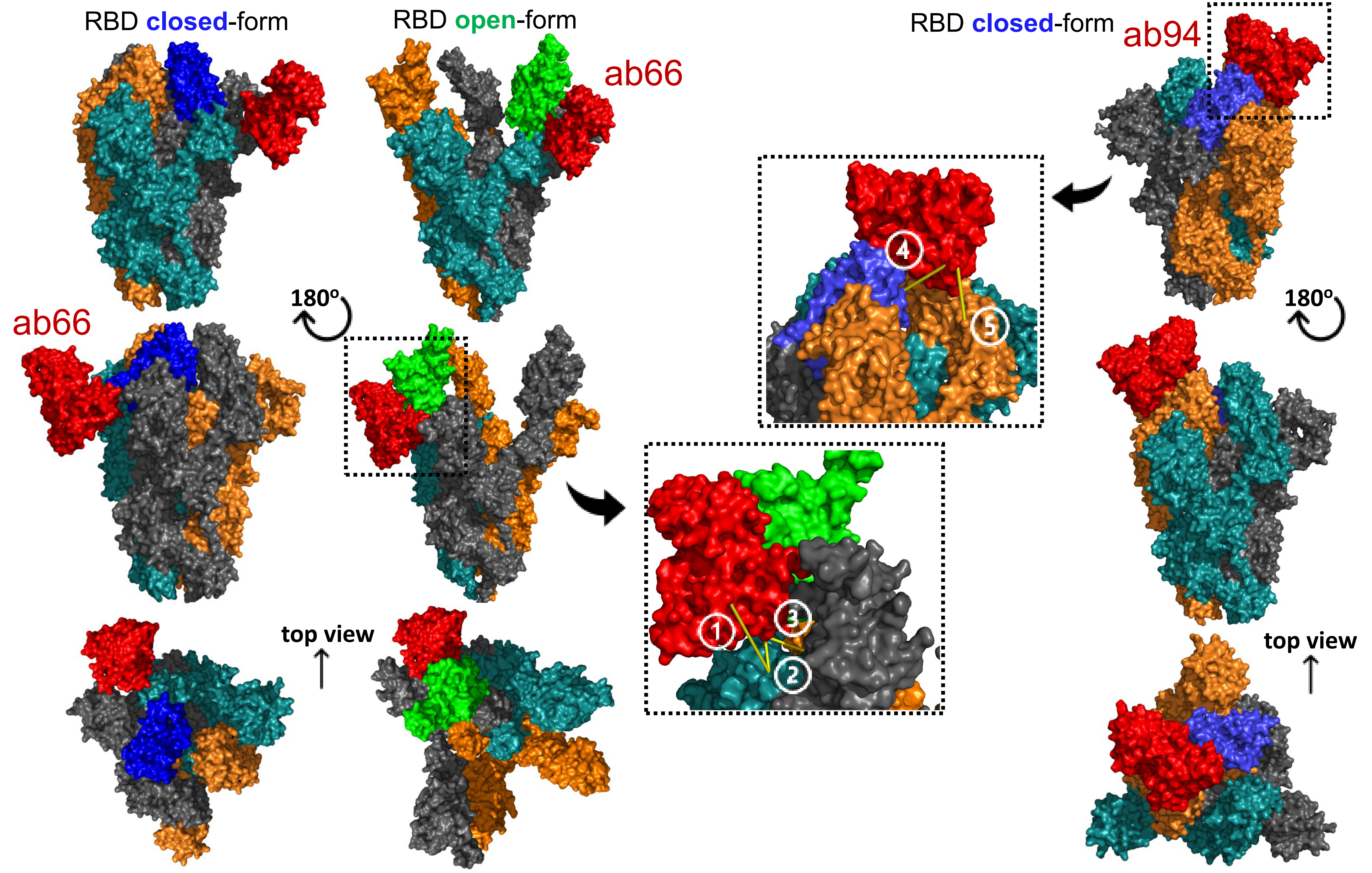

Supplementary Figure 3. Ab-Spike XL models highlight RBD binding. Ab66 could be modeled to both the closed and open form of RBD, whereas for Ab94 only models compatible with closed form was found.
