# Supplementary material for: Spike-Dependent Opsonization Indicates Both Dose-Dependent Inhibition of Phagocytosis and That Non-Neutralizing Antibodies Can Confer Protection to SARS-CoV-2
Source: Front Immunol. 2022 Jan 14;12:808932. doi: 10.3389/fimmu.2021.808932 (PMC8796240; doi:10.3389/fimmu.2021.808932)
Supplement: Supplementary file 4 [file DataSheet_4.pdf]

Supplementary Figure 4

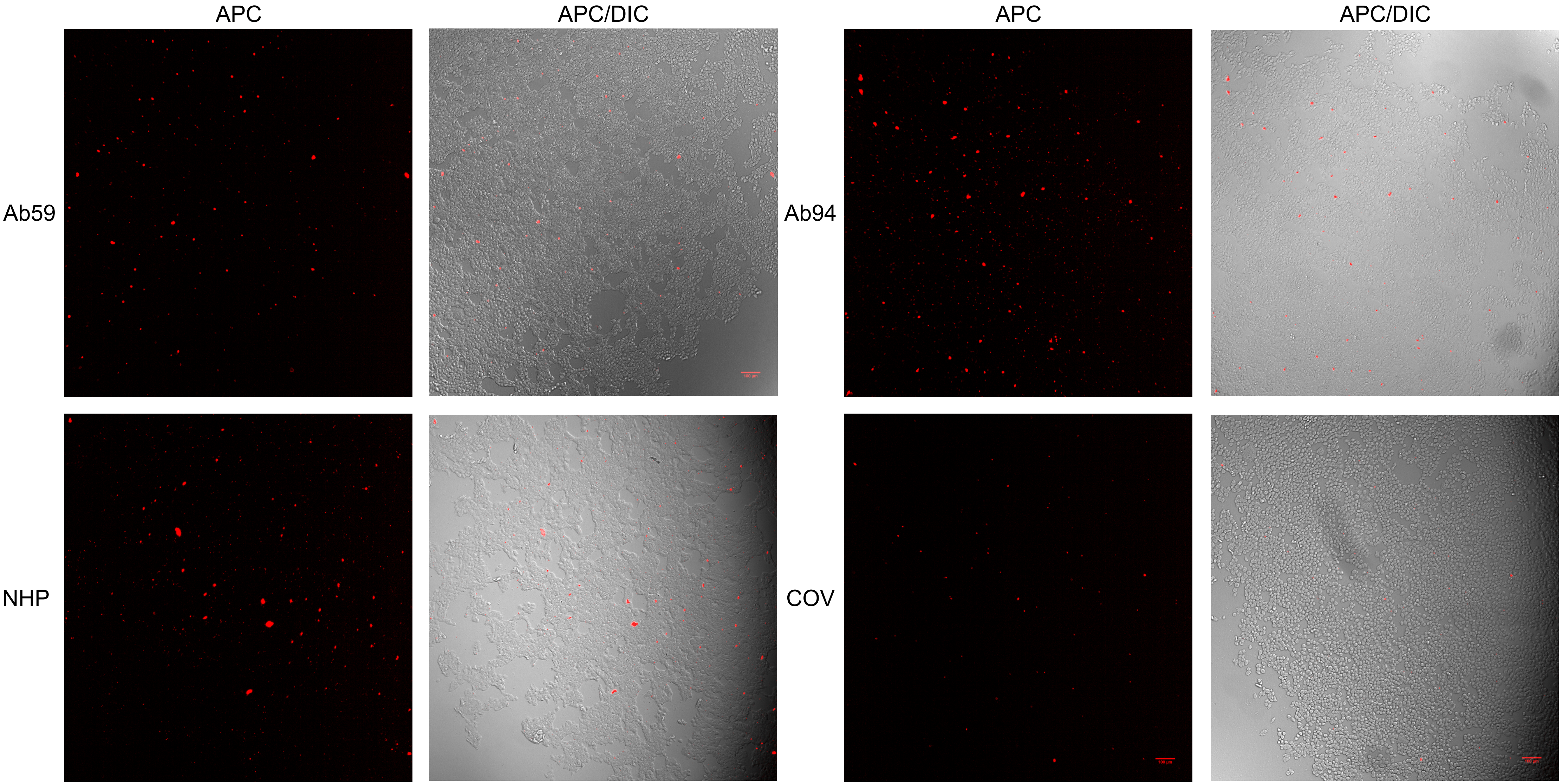

Supplementary Figure 4. Images from Spike-bead to ACE2+-cells binding neutralization experiments.

Representative images of the data collected and used to generate the bead-based neutralization results. Fluorescence (APC) images show beads and merged APC/DIC(10x) images for Ab59, 94, and control samples (normal human plasma and COVID19 patient plasma9. The scale bar represents a 100 µm distance.
