# Supplementary material for: Spike-Dependent Opsonization Indicates Both Dose-Dependent Inhibition of Phagocytosis and That Non-Neutralizing Antibodies Can Confer Protection to SARS-CoV-2
Source: Front Immunol. 2022 Jan 14;12:808932. doi: 10.3389/fimmu.2021.808932 (PMC8796240; doi:10.3389/fimmu.2021.808932)
Supplement: Supplementary file 5 [file DataSheet_5.pdf]

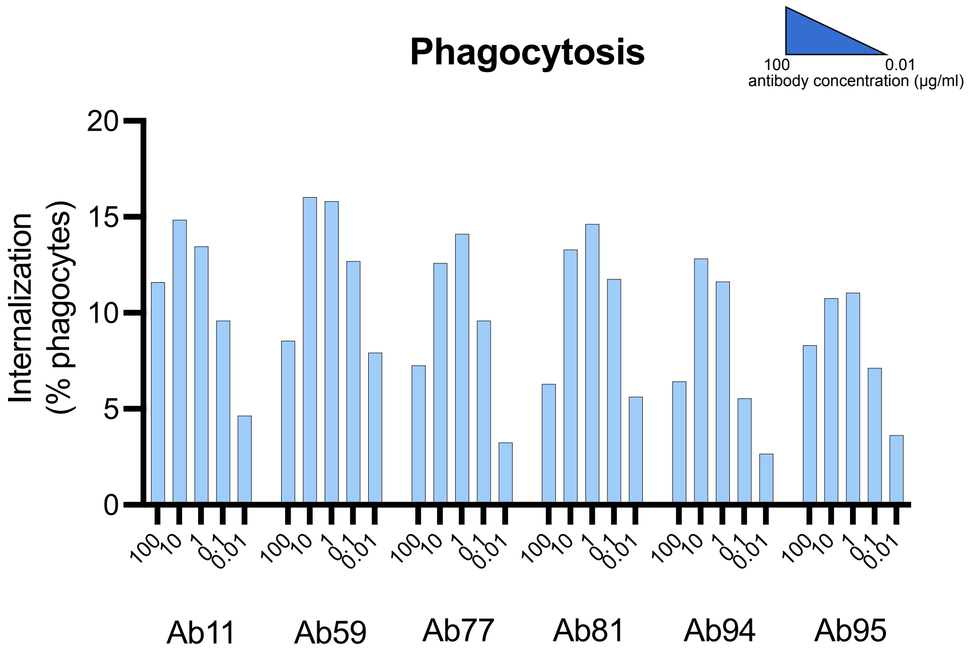

Supplementary Figure 5. Internalization control for phagocytosis experiments.

Spike beads were stained with the acid-sensitive dye pHrodo (FITC) and were then opsonized with dilutions of selected monoclonal antibodies. The cells were then analyzed by flow cytometry to determine the rate of bead internalization. A cell that was doubly fluorescent in APC and FITC has had internalized Spike-beads.
