# Supplementary material for: Spike-Dependent Opsonization Indicates Both Dose-Dependent Inhibition of Phagocytosis and That Non-Neutralizing Antibodies Can Confer Protection to SARS-CoV-2
Source: Front Immunol. 2022 Jan 14;12:808932. doi: 10.3389/fimmu.2021.808932 (PMC8796240; doi:10.3389/fimmu.2021.808932)
Supplement: Supplementary file 6 [file Table_1.pdf]

Table 1. Covid-19 patient data.

Patient	Clinical disease score	Age	Gender	Oxygen treatment
**1**	Mild	31	M	No
**2**	Moderate	53	M	Yes
**3**	Moderate	45	F	Yes
**4**	Mild	59	F	No
**5**	Mild	52	M	Yes
**6**	Moderate	53	M	No
**7**	Moderate	39	M	Yes
**8**	Moderate	49	F	No
**9**	Moderate	54	F	Yes
**10**	Moderate	34	M	Yes
**11**	Mild	75	F	No
**12**	Mild	30	M	No
**13**	Mild	71	F	No
**14**	Moderate	80	M	Yes
**15**	Severe	74	F	Yes
**16**	Severe	43	M	Yes
**17**	Moderate	54	F	Yes
**18**	Mild	60	F	No
**19**	Mild	59	M	No
**20**	Mild	72	M	No
